# Three-dimensional time-domain scattering of waves in the marginal ice zone

**DOI:** 10.1098/rsta.2017.0334

**Published:** 2018-08-20

**Authors:** M. H. Meylan, L. G. Bennetts

**Affiliations:** 1 School of Mathematical and Physical Sciences, University of Newcastle, Callaghan, New South Wales 2308, Australia; 2 School of Mathematical Sciences, University of Adelaide, Adelaide, South Australia 5005, Australia

**Keywords:** sea ice, wave scattering, marginal ice zone

## Abstract

Three-dimensional scattering of ocean surface waves in the marginal ice zone (MIZ) is determined in the time domain. The solution is found using spectral analysis of the linear operator for the Boltzmann equation. The method to calculate the scattering kernel that arises in the Boltzmann model from the single-floe solution is also presented along with new identities for the far-field scattering, which can be used to validate the single-floe solution. The spectrum of the operator is computed, and it is shown to have a universal structure under a special non-dimensionalization. This universal structure implies that under a scaling wave scattering in the MIZ has similar properties for a large range of ice types and wave periods. A scattering theory solution using fast Fourier transforms is given to find the solution for directional incident wave packets. A numerical solution method is also given using the split-step method and this is used to validate the spectral solution. Numerical calculations of the evolution of a typical wave field are presented.

This article is part of the theme issue ‘Modelling of sea-ice phenomena’.

## 1. Introduction

Vast areas of the polar ocean surface are frozen into a thin layer (centimetres to metres thick) known as sea ice. Sea ice plays an important role in the global climate, affecting heat fluxes between the atmosphere and ocean, and ocean circulation. Large-amplitude storm waves generated in the open ocean penetrate up to hundreds of kilometres into the sea ice-covered ocean—in the so-called marginal ice zone (MIZ)—and break up large ice floes (discrete chunks of sea ice) into floes with diameters comparable to the dominant wavelength. Modelling the propagation and attenuation of waves in the ice-covered ocean is relevant to the safe operation of ships [1] and climate science [2]. In recent years, technological advances, specifically the ability to deploy remote wave buoys that relay wave spectra, buoy position, etc., via satellite, has allowed accurate measurements over unprecedented scales [3,4]. These experimental results have shown the importance of wave effects.

There are two established paradigms for modelling attenuation of linear waves: wave scattering and dispersion relations [5]. Scattering models resolve the individual floes and are valid for large floes. They have been developed for over 20 years, beginning with [6], and have recently progressed to the point at which three-dimensional geometries involving realistic floe size distributions can be modelled [7,8], so that the waves spread directionally as well as attenuate [9]. Note that the waves are two dimensional as they exist at the ocean surface, with motions in the water column providing the third dimensions. Dispersion relation models treat the ice cover as a continuum (assuming small floes), with attenuation due to parametrized viscosity. They were originally proposed by Keller [10], and have recently been developed by, for example, Wang & Shen [11], including being tuned using field measurements [4]. While nonlinear effects can be included and are potentially important for large-amplitude waves [12], it is standard to assume linear motions.

Scattering is a three-dimensional phenomenon since the action of scattering is to redistribute wave energy directionally. However, two-dimensional scattering theory (i.e. one horizontal dimension and the vertical dimension) is the one which has found application in practical attenuation models. The first two-dimensional model was developed by Kohout & Meylan [13], and has been used been used in the WAM wave model [14] and the HYCOM ice–ocean model [15]. Similar models have been integrated into the most widely used community sea ice and wave models: respectively, CICE [16] and WAVEWATCH III [4]. Somewhat paradoxically, the first scattering models were three dimensional [17–20]. Large-scale wave prediction codes now require three-dimensional models to be implemented, primarily because of the need to include angular (directional) spreading. There are significant observations of angular spreading [21] and this effect has been shown to be important in recent three-dimensional simulations with large numbers of floes [7,8]. The three-dimensional scattering equation for wave propagation is given by a linear Boltzmann equation which models both dissipation and angular redistribution of wave energy. This model was independently proposed by Masson & LeBlond [17] and Meylan *et al.* [18], which were shown to be equivalent, after correcting errors in both models in [20]. The Boltzmann model was validated in [22] using laboratory experiments, and the diffusion limit for this model was considered in [23]. It should be noted that three-dimensional models require the solution of the three-dimensional scattering from an elastic ice floe and this is challenging to compute [24–27].

The linear Boltzmann equation is a classical model for a range of scattering phenomena. In nuclear physics, it models a low-density flow of neutrons [28]. It also often arises as a limiting case of energy transport in random media [29]. A method to solve the so-called Milne problem, in which the scattering is from a semi-infinite region with the solution isotropic in the transverse *y*-direction, was developed in [30]. Solutions for toy problems were computed, and an example of the kind of calculation which could give insights into scattering in the MIZ was given.

The present work aims to outline how the three-dimensional scattering of waves in the MIZ can be calculated in the time domain using a linear Boltzmann model. Section 2 presents the Boltzmann equation model. Section 3 shows how to compute the scattering from a single floe and how this leads directly to the kernel in the Boltzmann equation. In §4, we show how the solution for this Boltzmann equation in space and time can be found for the half-space problem. Section 5 presents some example calculations showing the evolution of a typical directional wave field. Section 6 is a brief conclusion. We also give an alternative time-marching solution method in appendix A.

## 2. Problem formulation

The wave energy balance equation is
2.1*a*
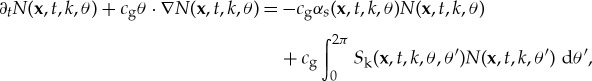
which is also known as the linear Boltzmann equation. Here, the Cartesian coordinate **x** = (*x*, *y*) denotes location on the ocean surface, ∇ = (∂_*x*_, ∂_*y*_) is the gradient operator, *t* is time, *N* is the so-called wave action density, *k* is the wavenumber (modulus of the wave vector), *θ* is the wave direction vector (argument of the wavenumbers in the *x*- and *y*-directions), *c*_g_ is the group velocity, *α*_*s*_ is the energy loss and *S*_k_ is the scattering kernel that redistributes wave energy in different directions. As scattering is an energy-conserving process, the redistribution of energy is exactly the loss of energy in the given wave direction, so that
2.1*b*
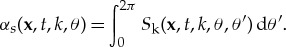
Non-conservative effects could easily be included by modifying the expression for *α*_*s*_.

We assume that the scattering is isotropic, in the sense that *S*(**x**, *t*, *k*, *θ*, *θ*^′^)≡*S*(**x**, *t*, *k*, *θ* − *θ*^′^), anticipating this condition will hold approximately and on average in a typical MIZ over sufficient spatial and/or temporal scales, as ice floes are orientated randomly. Equations (2.1) become
2.2*a*
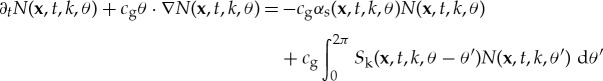
and
2.2*b*
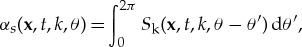
respectively. In this study, we achieve isotropic scattering by using modelling the ice floes as being circular. Non-circular models (e.g. [25,26,31]) could be used, but only with the additional burden of averaging the scattering characteristics.

## 3. Calculation of the scattering kernel

### (a) Solution for a circular floe

We briefly summarize the eigenfunction matching solution method proposed by Peter *et al*. [27] for a circular elastic plate model of an ice floe, floating on a three-dimensional water domain, which has finite depth and stretches to infinity in both horizontal directions. The plate has radius *a* and uniform thickness *h*, although varying thickness could be incorporated using the method of Bennetts *et al*. [32]. Assuming that all motions are time harmonic with radian frequency *ω*, the velocity potential of the water, 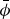, can be expressed as
3.1
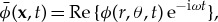
where the reduced velocity potential *ϕ* is complex-valued, and (*r*, *θ*, *z*) is a cylindrical polar coordinate system, assumed to have its origin at the centre of the circular plate. The reduced potential satisfies the boundary value problem
3.2
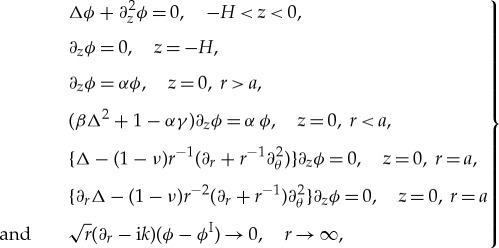
where
3.3
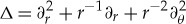
is the Laplacian operator in the horizontal plane, *k* is the open water wavenumber (defined below), *ϕ*^I^ is the incident potential, and *α* = *ω*^2^/*g* is a frequency parameter, in which *g* ≈ 9.81 m s^−2^ is the constant of gravitational acceleration. The parameters *β* and *γ* are
3.4
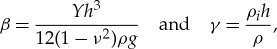
where *ρ* ≈ 1025 kg m^−3^ is the water density, *Y* ≈ 6 GPa is Young's modulus of the plate, *ν* ≈ 0.3 is its Poisson's ratio and *ρ*_*i*_ ≈ 992.5 kg m^−3^ is its density. The incident potential is set to be a plane wave with displacement amplitude *A*, travelling in the positive *x*-direction, i.e.
3.5
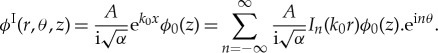


The potential is expanded in its eigenfunctions as
3.6
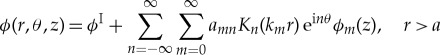
and
3.7
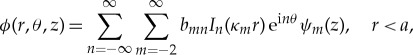
where *I*_*n*_ and *K*_*n*_ are modified Bessel functions of order *n*. The wavenumbers *k*_*m*_ and *κ*_*m*_ are the roots 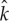 and 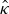 of the dispersion equations
3.8
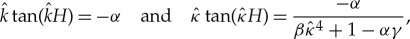
respectively [33]. The first dispersion relation has a unique purely imaginary solution in the upper half of the complex plane, denoted *k*_0_ = i*k*, and an infinite number of positive real solutions denoted *k*_1_ < *k*_2_ < · *s*. The second dispersion relation also has a unique purely imaginary solution in the upper half of the complex plane, *κ*_0_, and an infinite number of positive real solutions, *κ*_1_ < *κ*_2_ < · *s*. It also has two complex roots in the upper-half complex plane, denoted *κ*_−2_ and *κ*_−1_, where 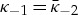. We define associated vertical eigenfunctions as
3.9
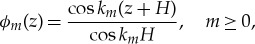
for the open water region, and
3.10
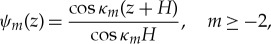
for the plate-covered region.

As the problem is axisymmetric, we can solve for the amplitudes *a*_*mn*_ and *b*_*mn*_ of each angular mode *n* separately, and we also note that the amplitudes for positive and negative *n* are complex conjugates so that solving for *n*≥0 is sufficient. For a given value of *n*, the amplitudes are obtained in the standard way, by matching the potential and its radial derivative at *r* = *a* for −*H* < *z* < 0, taking inner products with respect to *ϕ*_*m*_ (*m* = 0, 1, …), and truncating the resulting system of equations and all infinite sums to *m* ≤ *M*. The system is closed by applying the plate free edge conditions, which are the antepenultimate and penultimate conditions of equation (3.2).

### (b) Scattering kernel from the single-floe solution

Meylan *et al.* [18] gave the following justification for calculating the scattering kernel from the scattering produced by a single floe (based on the work of [29]). Each floe scatters energy, and the energy radiated per unit angle per unit time in the *θ* direction for a wave incident in the *θ*^′^ direction, *E*, is given by
3.11
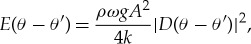
where *D*(*θ* − *θ*^′^) is the scattered amplitude. At a large distance from the scatterer, the asymptotic amplitude of the outgoing wave in the *θ* direction, for an incident wave travelling in the *θ*^′^ direction, is given by
3.12
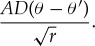


The scattering kernel, *S*_k_, is found by dividing *E* by the rate of energy passing under the ice floe. The rate of energy passing under the floe is given by the product of the wave energy density (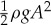), the effective area occupied by a floe (*A*_f_/*f*_i_, where *A*_f_ is the area of the floe and *f*_i_ is the fraction of the surface area of the ocean which is covered in ice), and the wave group speed (*ω*/2*k*). Thus,
3.13
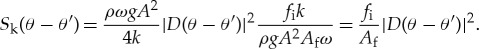


Using the asymptotic results for Bessel functions with large arguments, the far-field wave amplitude is
3.14
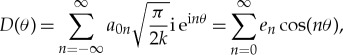
where we have used the property that the solutions for positive and negative *n* are complex conjugates. For the purposes of calculations in wave prediction code, values of *D* can be efficiently stored using only a small number of *e*_*n*_ values.

### (c) Identities

Here we derive identities for the solution of the circular ice floe problem, which are (to our knowledge) not known in the literature, and help validate our numerical results. The identities apply to the far-field calculation for anybody with circular symmetry. They follow from noticing that if the incident wave was a Hankel function of the second kind, then the outgoing wave at infinity would have to be a Hankel function of the first kind with the same amplitude (possibly with a different phase). As the incident wave is written as i^*n*^*J*_*n*_(*Kr*), it follows that
3.15
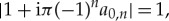
which means that
3.16
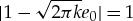
and
3.17
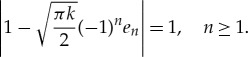


### (d) Numerical results for far-field scattering

**Figure 1.**
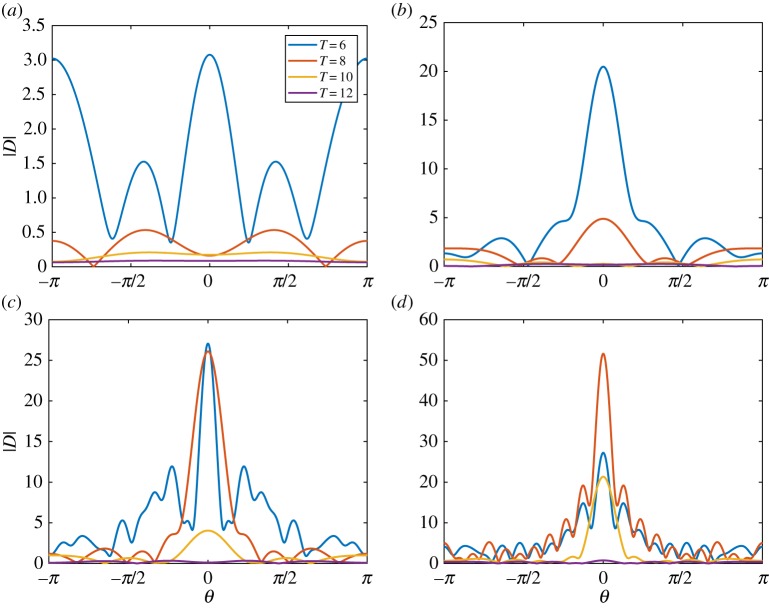
The absolute value of the far-field scattering |*D*(*θ*)| for an ice floe of radius *a* = 25 m (*a*), *a* = 50 m (*b*), *a* = 100 m (*c*) and *a* = 200 m (*d*). The floe thickness was 1 m. The periods were *T* = 6, 8, 10, 12 s as shown. (Online version in colour.)

Figure 1 shows the absolute value of the far-field scattering function, |*D*(*θ*)|, for a *h* = 1 m thick ice floe of radius (*a*) *a* = 25 m , (*b*) *a* = 50 m, (*c*) *a* = 100 m and (*d*) *a* = 200 m, and wave periods *T* = 6, 8, 10 and 12 s. This figure shows that the scattering is highly dependent on wave period, noting that from equation (3.13) the square of |*D*(*θ*)| controls the scattering of wave energy. As the floe radius increases, the scattering becomes more complicated with respect to angle, but given the variation in floe size in an MIZ this effect will be removed by averaging. The scattering is strongest in the *θ* = 0 direction, which is related to the so-called shadow zone behind the floe, where the scattered wave almost cancels the incident wave.

**Figure 2.**
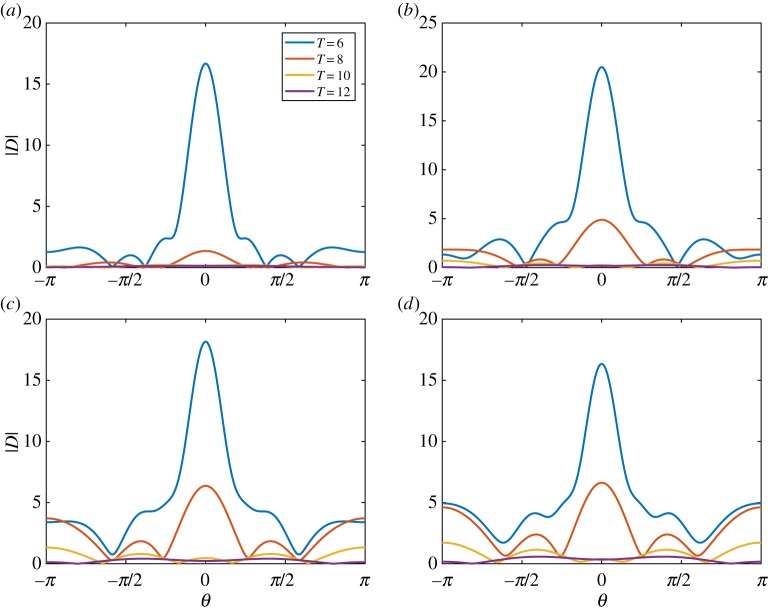
The absolute value of the far-field scattering |*D*(*θ*)| for a floe of thickness (*a*) *h* = 0.5 m, (*b*) *h* = 1 m, (*c*) *h* = 1.5 m and (*d*) *h* = 2 m. The floe is of radius *a* = 50 m, and wave periods are *T* = 6, 8, 10 and 12 s. (Online version in colour.)

**Figure 3.**
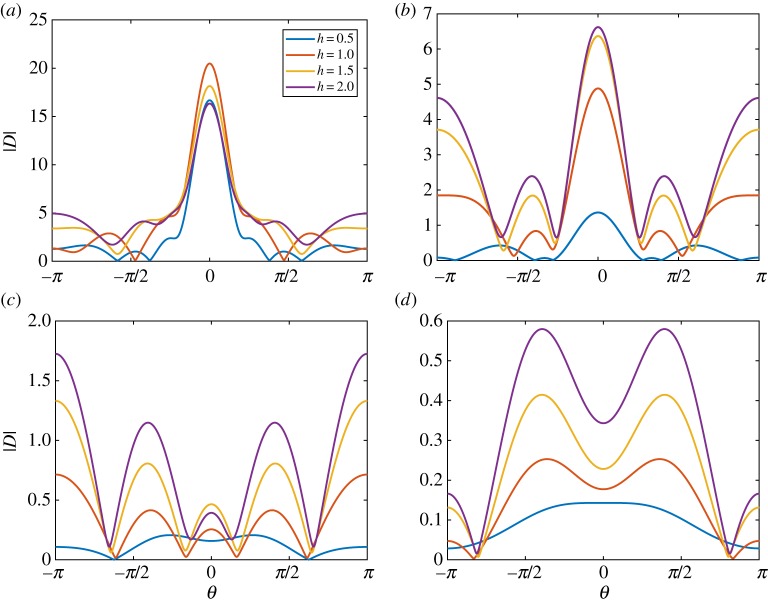
The absolute value of the far-field scattering |*D*(*θ*)| for a wave of period (*a*) *T* = 6 s, (*b*) *T* = 8 s, (*c*) *T* = 10 s and (*d*) *T* = 12 s. The floe is of radius *a* = 50 m, and thicknesses *h* = 0.5, 1.0, 1.5 and 2.0 m. (Online version in colour.)

Figure 2 shows |*D*(*θ*)| for an ice floe of thickness (*a*) *h* = 0.5 m, (*b*) *h* = 1 m, (*c*) *h* = 1.5 m and (*d*) *h* = 2 m. The floe radius is *a* = 50 m, and the wave periods are *T* = 6, 8, 10 and 12 s. The figure shows that variations in floe thickness affect the scattering for small thicknesses, but, above a critical thickness, scattering becomes insensitive to thickness changes. This insensitivity is because the floe bending is negligible above the critical thickness. The critical thickness is period dependent and increases as the period increases. The behaviour is also illustrated in figure 3, where we vary the thickness rather than the period in each subplot.

**Figure 4.**
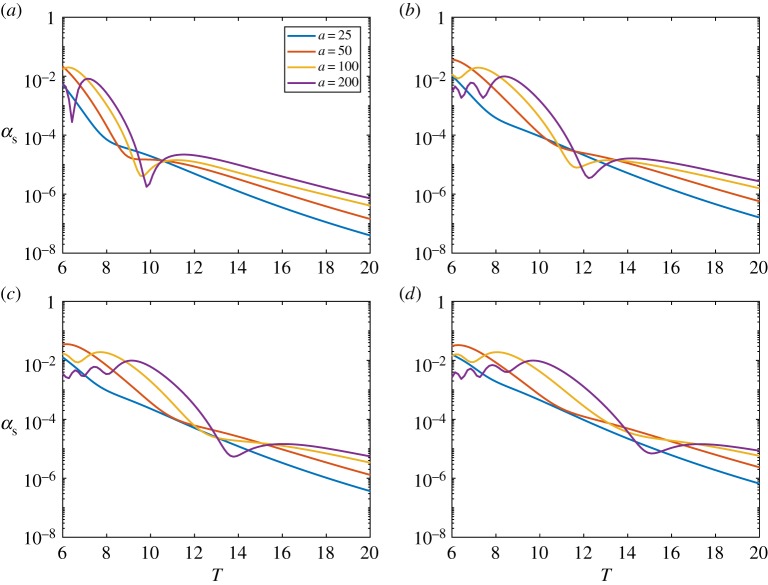
The parameter *α*_*s*_ as a function of period *T* for the floe radii shown. The thickness was (*a*) *h* = 0.5 m, (*b*) *h* = 1.0 m, (*c*) *h* = 1.5 m and (*d*) *h* = 2.0 m. (Online version in colour.)

It is informative to plot *α*_*s*_, as it determines the strength of the scattering. Note that, if all the scattered wave energy was removed from the system, then the wave field would decay as e^−*α*_*s*_*x*^. Figure 4 shows *α*_*s*_ as a function of wave period for various thicknesses and floe radii. The plot is on a log scale in *α*_*s*_, and shows that the scattering depends strongly on period but only weakly on the other parameters. The sharp dips indicate resonances and would be removed by averaging with respect to the floe properties. The figure shows that the wave period has the strongest control over scattering.

## 4. Temporal solution of the half-space scattering problem

Consider the half-space scattering problem for the wave energy balance equation (2.2), in which *S*_k_ = 0 for *x* < 0, and *S*_k_ = *S*_k_(*t*, *k*, *θ* − *θ*′)≠0 for *x* < 0, i.e. open water exists for *x* < 0 and a spatially homogeneous MIZ exists for *x* > 0. Further, we assume that the solution is independent of the *y*-coordinate.

### (a) Transformation to a discrete problem

We approximate the integral operator appearing in the wave energy balance equation (2.2) using 2*n* angular values

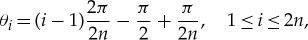
where *n* is odd to ensure that we do not have *θ*_*i*_≠ ± *π*/2, which would cause singularities. (A modified solution method for this case is given by Meylan [30].) Equation (2.2a) becomes
4.1
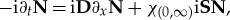
where

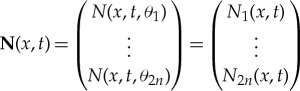


is the discrete intensity, *χ*_*Γ*_(*x*) is the characteristic function, which is zero for *x*∉*Γ* and unity for *x*∈*Γ*, and **D** is a diagonal matrix, which is self-adjoint but not positive, and **S** is a positive self-adjoint matrix. The entries of **D** and **S** are, respectively,
4.2
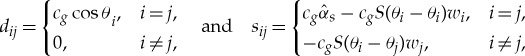
where *w*_*i*_ is the weight associated with the chosen quadrature rule (we use the trapezium rule so *w*_*i*_ = *π*/*n*) and
4.3
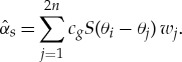
Symmetry means 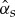 is independent of *i* and 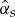 is a numerical approximation of *α*_*s*_ consistent with our discretization, ensuring energy is conserved. Further, the matrix **S** is a circulant matrix.

### (b) Non-dimensionalization

Non-dimensionalizing the problem makes the numerical scheme more stable, and, as will be shown, the time and length scales we use control the gross behaviour of the solution. We scale the temporal coordinate with respect to 1/*s*_11_. For a large number of angles (*n*≫1), we note that 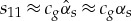, which is the exponential decay rate in time in the case that all scattered wave energy is lost. We scale the spatial coordinate so that in the non-dimensional problem the group velocity is unity. Therefore, we transform time and space as
4.4
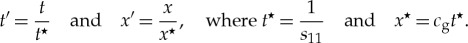
For a large number of discrete angles, 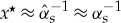, which is the length scale of the exponential decay in space if all the scattered waves are removed. Under this non-dimensionalization, equation (4.1) becomes
4.5
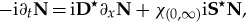
where the entries of **D**^⋆^ and **S**^⋆^ are
4.6
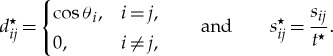
Thus, the entries of the matrices vary between −1 and 1, and we have *s*^⋆^_*ii*_ = 1. This makes the numerical schemes stable and the same discretization can be used in space and in time. We will drop the star from now on.

### (c) Solution using scattering theory

The solution of equation (4.5) can be written in abstract form as
4.7
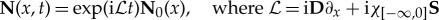
is an operator that defines a continuous semi-group, for which Meylan [30] outlined a method to diagonalize. Here, we focus on calculations of the spectrum of the operator 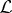 to show how important this is for controlling wave scattering. We note that if 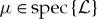, then 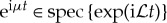. We will show how to compute this semi-group using a modified scattering theory, for the case of an incident wave group, which is initially zero in the MIZ (*x* > 0).

The spectrum of 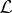 is defined by
4.8
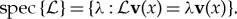
Separating the spectrum in the two regions *x* > 0 and *x* < 0, we obtain
4.9
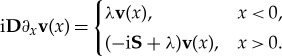
Solving, we obtain
4.10
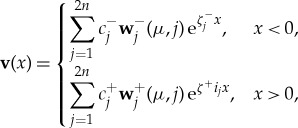
where **w**^−^_*j*_ are the eigenvectors of the matrix −i**D**^−1^λ and *ζ*^−^_*j*_ are the corresponding eigenvalues, and **w**^+^_*j*_ are the eigenvectors of the matrix −i**D**^−1^( − i**S** + λ) and *ζ*^+^_*j*_ are the corresponding eigenvalues. The constants *c*^±^_*j*_ are at this stage arbitrary.

At first sight, it appears that there will be a solution of (4.8) for every value of λ. However, we need to impose the condition that the solution is bounded at *x* = ± ∞. From symmetry, in both cases the eigenvalues come in plus and minus pairs, meaning we can eliminate half the eigenvectors and this leaves us only 2*n* unknowns. Matching at *x* = 0, we obtain 2*n* homogeneous equations, for which there will only exist a trivial solution. However, if either *ζ*^−^_*j*_ or *ζ*^+^_*j*_ is purely imaginary, we will have one extra unknown (as we can keep both the plus and minus solutions). As the number of unknowns is one greater than the number of homogeneous equations, we will have a non-trivial solution. This non-trivial solution is determined up to a constant and this will lead to the spectrum of the operator 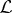. The corresponding functions for the eigenvalues of 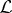 are known as generalized eigenfunctions.

For 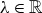, the solution for *x* < 0 gives 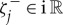. We denote the corresponding generalized eigenfunctions (with multiplicity *n*) by **v**^−^_*i*_, where

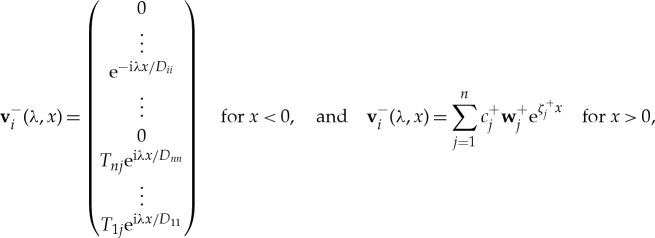
where the summation for *x* > 0 is over eigenvalues with negative real part only, i.e. Re(*ζ*^+^_*j*_) < 0. The generalized eigenfunctions **v**^−^_*i*_ are chosen to be non-zero only in a single incoming direction, which makes subsequent calculations easier. The *n* equations obtained by matching the solution for the first *n* rows at *x* = 0 uniquely determine the coefficients *c*^+^_*j*_. Subsequently, by matching the second *n* rows at *x* = 0 the elements of the matrix *T*_*ij*_ are found.

The complex spectrum of 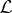 consists of λ such that
4.11
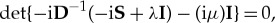
where **I** is the identity matrix, for a given 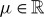. Equation (4.11) can be solved by finding the eigenvalues of the matrix −*μ***D** + i**S** for 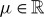. Therefore, the complex spectrum consists of *n* lines in the complex plane, which lie in the upper-half plane so that the semi-group is a contraction.

The generalized eigenfunctions for the complex spectrum are zero in the incident directions, so that energy is allowed to leave the MIZ only. The eigenfunctions are **v**^+^_*j*_, where

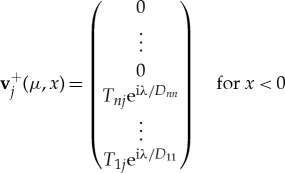
and

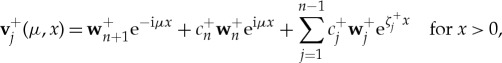
where, as before, the summation for *x* > 0 is over eigenvalues with negative real part only, i.e. Re(*ζ*^+^_*j*_) < 0. As the ordering of the eigenvectors for *x* > 0 is arbitrary, we choose, without loss of generality, that eigenvectors corresponding to i*μ* and −i*μ* have numbering *n* and *n* + 1, respectively. Further, we choose the values of *c*^+^_*j*_ so that the solution is zero in the incoming directions. The elements of the matrix *T*_*ij*_ are then found by matching the second *n* rows at *x* = 0.

**Figure 5.**
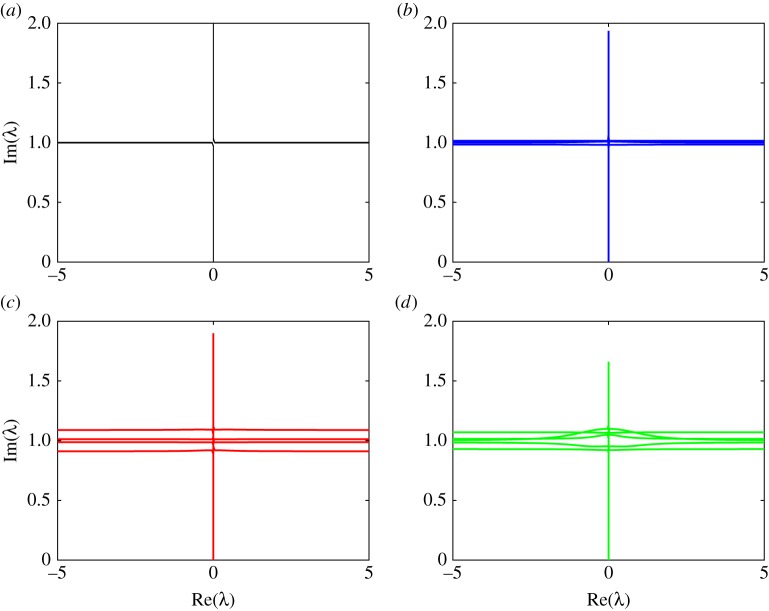
The branches of the complex spectrum for a wave period *T* = 8 s, and floe of thickness *h* = 1 m and radius *a* = 50 m, for the number of angles 2*n* shown. The solution is plotted in non-dimensional space. (*a*) 2*n* = 4, (*b*) 2*n* = 6, (*c*) 2*n* = 8 and (*d*) 2*n* = 10. (Online version in colour.)

**Figure 6.**
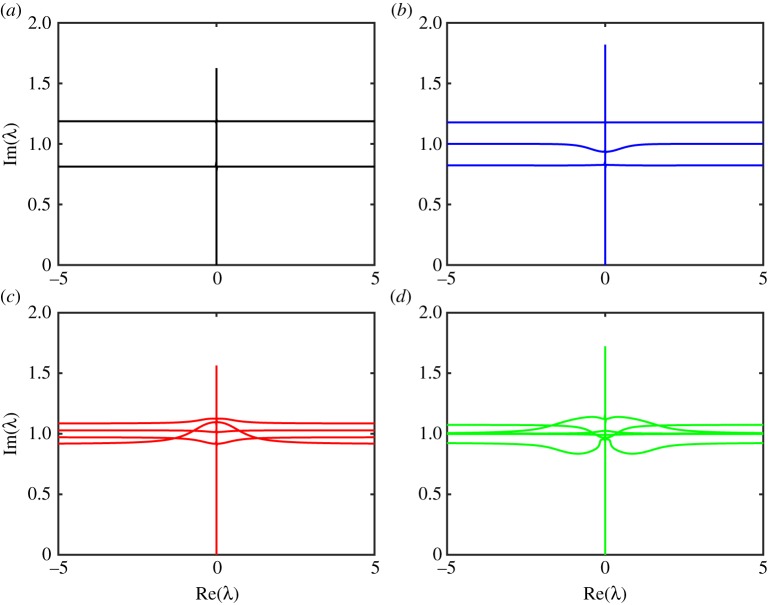
The branches of the complex spectrum for a wave period *T* = 12 s, and floe of thickness *h* = 1 m and radius *a* = 200 m, for the number of angles 2*n* shown. The solution is plotted in non-dimensional space. (*a*) 2*n* = 4, (*b*) 2*n* = 6, (*c*) 2*n* = 8 and (*d*) 2*n* = 10. (Online version in colour.)

Figures 5 and 6 show the branches of the spectrum of the operator 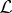 for different values of the angle truncation parameter *n*. Figure 5 shows the spectrum for a floe of thickness *h* = 1 m and radius *a* = 50 m, and wave period of *T* = 8 s. Figure 6 shows the spectrum for a floe of thickness *h* = 1 m and radius *a* = 200 m, and wave period of *T* = 12 s. These figures are the only plots we present in non-dimensional variables and the reason for this is the following. The imaginary part of this spectrum represents the decay which is proportional to the diagonal element of **S** since decay in this context is spreading. As the non-dimensional diagonal is always unity, we see that the complex spectrum consists of a vertical branch and lines which all have imaginary part ≈ 1. This pattern of the spectrum implies that the parameters *t*^⋆^ and *x*^⋆^ we calculate control the properties of the solution in time and space, respectively. It is interesting to ask what happens in the limit as the number of angles tends to infinity. This limit should allow us to compute the spectrum of the continuous operator which appears in equation (2.2a). Perhaps the spectrum collapses to a single line, or perhaps it fills a region of the complex plane.

We construct the solution to the time-dependent half-space problem using a version of the method developed by Meylan [30], which is based on the generalized eigenfunctions **v**^−^_*i*_. For an incident wave which is initially zero in the MIZ, it is possible to use only the real part of the spectrum of 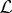 and only **v**^−^_*i*_. Physically, these are the only cases which could be excited, but mathematically we could start with the initial condition non-zero in the MIZ. We assume the initial wave action density in the MIZ is zero, i.e. **N**_0_(*x*) = 0 for *x* > 0, and that it is of the form
4.12
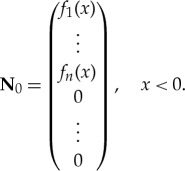
Therefore, we represent **N**_0_ in terms of the eigenfunctions **v**^−^_*i*_(λ, *x*)
4.13
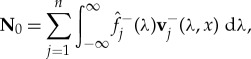
and, using the properties of the Fourier transform, we deduce that
4.14
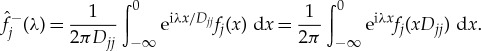
Note that the functions 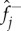 are not exactly the Fourier transform of *f*_*j*_ because of the *D*_*jj*_ term. Therefore, the solution is
4.15
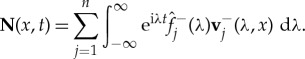
The integrals in equations (4.14) and (4.15) are calculated using the fast Fourier transform.

### (d) Validation via numerical method

**Figure 7.**
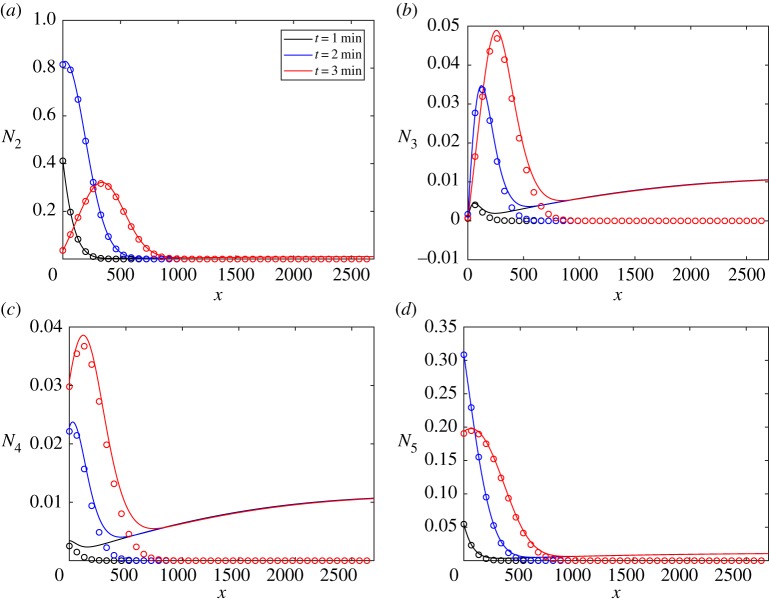
A comparison of the solution calculated by the scattering theory (solid line) and the split-step method (dots) for the times shown for the wave action density in the channels shown. The incident wave is of period *T* = 8 s, radius *a* = 50 m and thickness *h* = 1 m. The initial condition is given by equation (4.16). The number of angles is 6. (*a*) *n* = 2, (*b*) *n* = 3, (*c*) *n* = 4 and (*d*) *n* = 5. (Online version in colour.)

To validate the above spectral method based on scattering theory, we solve equation (4.5) using a so-called split-step method, described in appendix A. Figure 7 shows the evolution of the wave action density calculated using scattering theory and the split-step method. The incident wave field is uni-directional, travelling in the direction of the *x*-axis and with wave period *T* = 8 s, and the MIZ contains floes of radius *a* = 50 m and thickness *h* = 1 m. We consider the case when there are 2*n* = 6 angles, which is unlikely to be sufficient for practical calculations but is enough to validate the scattering theory. The initial wave profile is the Gaussian
4.16
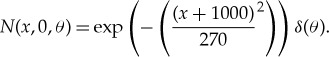
The agreement is generally excellent, with only small deviations at large distances, as the fast Fourier transform component of the spectral method is periodic, resulting in a residual solution, evident in figure 7*b*,*c*.

## 5. Numerical results

Numerical results are given for floe radius *a* = 50 m and thickness *h* = 1 m, an incident wave field with period *T* = 8 s, and shape
5.1
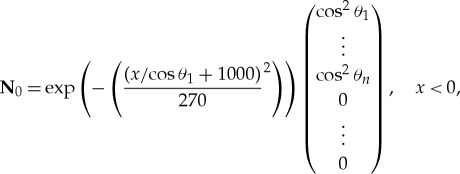
as given by Longuet-Higgins [34]. This form is chosen so that the intensity at the edge of the scattering region gives uni-modal (in angle) input in the direction moderated by a Gaussian in space, i.e.

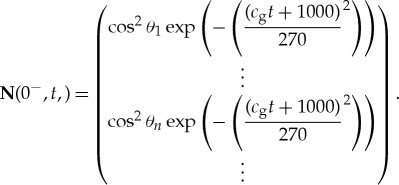


**Figure 8.**
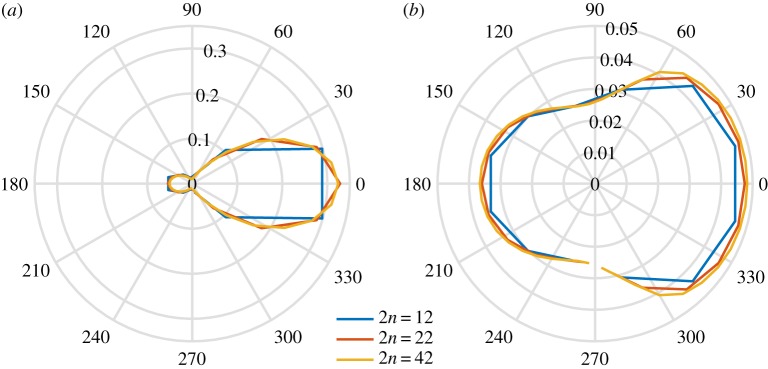
Polar plots of the solution for (*a*) 500 m and 4 min and (*b*) 1000 m and 8 min, for the number of angles shown for a period *T* = 8 s, radius *a* = 50 m and thickness *h* = 1 m. (*a*) *x* = 500 m, *t* = 4 min and (*b*) *x* = 1000 m, *t* = 8 min. (Online version in colour.)

**Figure 9.**
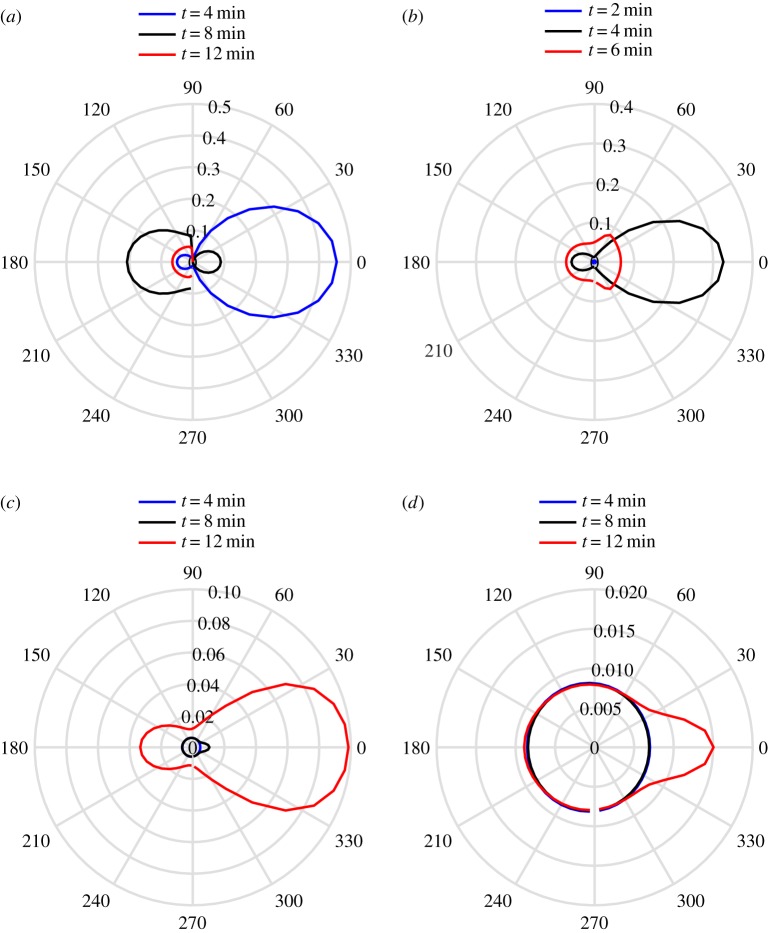
Polar plots of the solution for an initial condition given by equation (5.1) for (*a*) *x* = 0 m, (*b*) *x* = 500 m, (*c*) *x* = 1000 m and (*d*) *x* = 1500 m, for wave period *T* = 8 s, radius *a* = 50 m and thickness *h* = 1 m. (Online version in colour.)

**Figure 10.**
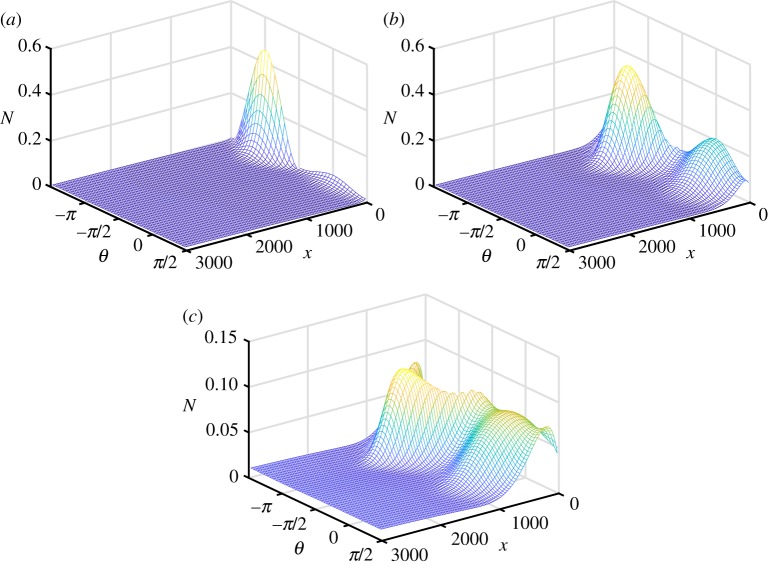
Mesh plot of the solution for (*a*) 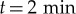, (*b*) 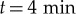 and (*c*) 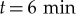, for wave period *T* = 8 s, radius *a* = 50 m and thickness *h* = 1 m. (Online version in colour.)

Figure 8 shows convergence plots for the solutions calculated with 2*n* = 12, 22 and 42, with a converged solution achieved for 2*n* = 42. This plot shows that the solution is well converged for 2*n* = 42, noting that this is more angles than are normally used in global wave prediction codes. On this basis, figure 9 shows the evolution of polar plots for 2*n* = 42. It is apparent that the amplitude of the scattered field rapidly decreases with distance into the MIZ and that the wave field becomes isotropic. Figure 10 shows corresponding three-dimensional mesh plots of the wave field as functions of wave direction.

## 6. Conclusion

We have investigated a Boltzmann equation model for wave scattering in the MIZ. We have shown how to derive the scattering kernel appearing in the Boltzmann equation from the solution for a single circular ice floe and presented new identities to validate it. We developed a mathematical theory to analyse the structure for the half-space problem under discretization in angle. We calculated the spectrum of the linear operator and showed that under a particular non-dimensionalization it has universal properties. We showed that a solution can be found using a scattering theory for incident waves, and validated the spectral solution against a split-step method. The method allows computation of the spatial and temporal evolution of wave packets propagating through the MIZ.

## Supplementary Material

Matlab code to produce the figures

## Data accessibility

The electronic supplementary material contains the Matlab code to calculate all the figures we have presented.

## Appendix A. Time marching solution method

We derive here a time-stepping solution method. We begin with the discrete Boltzmann equation (4.5), where we will exploit our non-dimensionalization in this numerical scheme. The equation is

A 1

(A 1) can be solved by a very simple time-stepping method known as the split-step method. For small time steps Δ*t*, we can approximate  as follows:

A 2

The action of these two operators separately is simple. The operator e^iΔ*t*/2**D**(∂/∂*x*)^ is simply a shift by an amount *D*_*jj*_Δ*t*/2, which acts on each channel separately, i.e.

Note that because we apply this shift operator as the first and last step we will be calculating e^iΔ*t*/**D**(∂/∂*x*)^ except for the first and last step. This is analogous to the trapezium method in numerical integration. We have non-dimensionalized so that the entries of **D** are between −1 and 1. This means that if we discretize the space into points spaced equally apart with spacing Δ*x*, then we can set Δ*t* = Δ*x* and we can approximate as follows:

where we are assuming that *D*_*jj*_ is positive. The negative case we change from *i* − 1 to *i* + 1, etc. We note that this time-stepping part of the equation is highly optimized in code such as WAVEWATCH III^®^.

The matrix exponential e^−*χ*_(0,∞)_**S**Δ*t*^ can be calculated from the eigenvectors of **S**. We know that the eigenvectors have a very simple structure from circulant matrix theory and they are given by

A 3

where superscript ‘T’ denotes transpose and  are the 2*n*th roots of unity. The eigenvectors **w**_*j*_ are orthonormal. The corresponding eigenvalues are then given by

where *s*_*ij*_ are the elements of **S**. Therefore, we can calculate e^−*χ*_(0,∞)_**S**Δ*t*^ as

Note this expression would be very useful in global wave prediction codes such as WAVEWATCH III^®^ if scattering by ice floes was to be included.
